# Panax notoginseng saponins promote liver regeneration through activation of the PI3K/AKT/mTOR cell proliferation pathway and upregulation of the AKT/Bad cell survival pathway in mice

**DOI:** 10.1186/s12906-019-2536-2

**Published:** 2019-06-10

**Authors:** Hua Zhong, Hao Wu, He Bai, Menghao Wang, Jian Wen, Jianping Gong, Mingyong Miao, Fangchao Yuan

**Affiliations:** 1 Department of Hepatobiliary Surgery, the First Affiliated Hospital of Chongqing Medical University, Chongqing, 400010 China; 2 Department of Hepatobiliary Surgery, the Second Affiliated Hospital of Chongqing Medical University, Chongqing, 400000 China; 3 Department of Biochemistry and Molecular Biology, Second Military Medical University, 800 Xiangyin Road, Shanghai, 200433 China

**Keywords:** *Panax notoginseng* saponins, Liver regeneration, Protein kinase B, Mammalian target of rapamycin

## Abstract

**Backgroud:**

The regenerative capacity of the liver is crucial for the host to survive after serious hepatic injuries, tumor resection, or living donor liver transplantation. *Panax notoginseng* saponins (PNS) have been reported to exert protective effects during organ injuries. The present study aimed to evaluate the effect of PNS on liver regeneration(LR) and on injuries induced by partial hepatectomy (PH).

**Methods:**

We performed 70% partial PH on C57BL/6 J mice treated with or without PNS. LR was estimated by liver weight/body weight, serum alanine aminotransferase (ALT) and aspartate aminotransferase (AST) levels and cell proliferation, and the related cellular signals were analyzed by Western blot.

**Results:**

Different concentrations of PNS promoted hepatocyte proliferation in vitro. Mice in the PNS group showed higher liver/body weight ratios at 2 d and 7 d (*P* < 0.05) after PH and lower levels of serum ALT and AST (*P* < 0.05) compared to those of mice in the normal control (NC) group. Histological analysis showed that the expression of proliferating cell nuclear antigen(PCNA) at 2 d and 7 d after PH was significantly higher in the PNS group than in the NC group (*P* < 0.05). Mechanistically, the AKT/mTOR cell proliferation pathway and AKT/Bad cell survival pathway were activated by PNS, which accelerated hepatocyte proliferation and inhibited apoptosis (*P* < 0.05).

**Conclusions:**

PNS promoted liver regeneration through activation of PI3K/AKT/mTOR and upregulated the AKT/Bad cell pathways in mice.

## Backgroud

The liver, which has a remarkable regenerative capability, is able to fully regenerate to its original mass, even with less than 30% of its tissue left [1]. This regenerative capacity is crucial for the host to survive after serious hepatic injuries, tumor resection, or living donor liver transplantation. Liver function recovery depends on increased energy production, which is indispensable for restoring liver mass [2]. However, the mechanisms underlying the response of the liver to acute and chronic injury and signaling pathways that are critical for regenerative capacity still require further study.

The traditional Chinese medicinal herb *Panax notoginseng*(Sanqi), which is known as a hemostatic medicine, has been used to accelerate blood clotting, alleviate swelling and relieve pain for more than a thousand years [3]. *Panax notoginseng* saponins (PNSs)contains more than 20 dammarane type saponins including ginsenoside Rg1, Rg2, Rb1, Rb2, Rb3, Rc, Rd., Re, Rh, F2 and notoginsenoside R1, R2, R3, R4, R6, Fa, Fc, Fe, etc., among which *panax* notoginsenoside R1, ginsenoside Rg1, Rd., Re and Rb1 are the major active components [4–6]. More than 200 compounds have been isolated from PNS, including saponins, flavonoids, cyclopeptides, sterols, polyacetylenes, volatile oil, amino acids, and polysaccharides [7]. Various PNS preparations are commercially available and widely applied in pharmaceutical forms, including capsules, injections, and powders [8]. Increasing evidences has indicated that PNS can not only prevent the aggregation of platelets to invigorate the circulation of blood but can also stimulate angiogenesis to repair injured vessels and recover the blood supply for different organs [9]. In addition, PNS prevents cardiomyocyte apoptosis and alleviates oxidative stress-induced damage [10]. PNS protects human umbilical vascular endothelium cell against oxidative damage in vitro [11]. Moreover, PNS has been used in liver diseases. PNS ameliorates liver fibrosis in vivo and vitro [12]. Zhou’s study found that PNS protected liver against hepatotoxicity induced by Triptolide [13]. The protective effect of PNS against liver injuries in mice induced by ethanol has been verified in previous study [14]. Hepatic hemodynamics changed obviously and oxidative stress were increased after partial hepatoectomy (PH) and a quick recover of blood supply and relieved oxidative stress in liver would be beneficial for liver regeneration [15]. It is reasonable to predict that PNS may have protective effects against injuries induced by PH. However, the effect of PNS on the injury induced by PH has never been studied.

The phosphatidylinositol-3-kinase (PI3K)-Protein kinase B(AKT) signaling pathway has long been confirmed as an important pathway regulating cell proliferation. Phosphorylated AKT acts on its downstream targets and participates in the regulation of the cell cycle and growth [16]. It has been reported that PNS activated the PI3K/AKT/mammalian target of rapamycin(mTOR) signaling pathway in anterior cruciate ligament fibroblasts [17]. However, whether the PI3K/AKT signaling pathway in primary hepatocytes is activated upon treatment with PNS is unknown.

The present study examined the effect of PNS on 70% liver resection of liver regeneration (LR) mouse models and studied the underlying mechanism.

## Methods

### Experimental animals

Male C57BL/6 mice that weighed 19–23 g and were 8–10 weeks of age were purchased from the Experimental Animal Center of Chongqing Medical University (Chongqing, China). Animals were housed in sterile polycarbonate cages with free access to water and food in a specific pathogen-free (SPF) barrier. All animal care and experimental protocols were in compliance with the Animal Management Rules of the Ministry of Health of the People’s Republic of China.

### Animal surgery

PNS were purchased from Kunming Pharmaceutical Corporation(China Food and Drug Administration (CFDA) approval number: Z20026438, KPC Pharmaceuticals, Inc., Kunming, China). Seven days before PH, mice were divided into two groups that were administered saline (normal control, NC) or PNS (200 mg/kg, intraperitoneal injection) at 8:00 am everyday [18]. The treatment is maintained during the whole experiment. The same surgical procedure were performed in both NC and PNS group. Before PH, mice were subjected to adequate anesthesia by sodium pentobarbital (50 mg/kg) intraperitoneal injection while the body temperature was sustained by a heating pad at around 36 °C. Two-thirds of the liver (median and left lobes) was removed [19]. All surgeries were performed at 9:00–11:00 AM to rule out the influence of diurnal variations on regeneration. Then, the mice were divided into 3 groups (*n* = 6 animals per group): PH 2 d, 7 d and sham-operation (sham) groups. The same surgical procedure was performed on the sham groups without conducting PH. The mice were anaesthetized by sodium pentobarbital (50 mg/kg) intraperitoneal injection and sacrificed by cervical dislocation, and the remnant liver tissues were harvested at different time points: 2 and 7 days (s). The regenerative remnant lobe was snap-frozen in liquid nitrogen and stored at − 80 °C for further analysis.

### Isolation of primary hepatocytes and cell culture

Primary hepatocytes were isolated according to a method introduced by Edwards M et al. [20]. Briefly, under adequate anesthesia, the inferior vena cava (IVC) was exposed. The IVC was infused with buffer 1 (Ca^2+^- and Mg^2+^-free Hanks balanced salt solution (HBSS)) after it was blocked just below the heart. The infusion was changed to buffer 2 (Ca2 + − and Mg2 + −containing HBSS to a final concentration of 0.08 U/mL) and the perfusion was continued for approximately 8–10 min after perfusing buffer 1 for 8 min (5 mL/min). The liver was then placed in a 100-mm cell-culture plate for further hepatocyte isolation. Finally, 1 mL (5 × 10^6^ hepatocytes/mL) of the suspension was dispensed into each well of 12-well culture plates and incubated at 37 °C with 5% CO_2_ for later use. Hypoxia was simulated according to the study design by Hong et al. [21]. Hepatocytes were preincubated with 10 μM LY294002 for 1 h and were then treated with PNS (0.12 mg/ml) for 24 h. Six hours after the addition of PNS (0.12 mg/ml), the cells were subjected to hypoxia for 24 h.

### Cell viability

Primary mouse hepatocytes (6 × 103 cells/well) were treated with a variety of concentrations of PNS for 24 h in 96-well plates. Cell viability was determined by a Cell Counting Kit-8 (MCE, Prod# HY-K0301) according to the company’s protocol. The absorbance at 450 nm was measured by a microplate reader (xMark™ Microplate Absorbance Spectrophotometer, BIO-RAD). Values represent the mean ± SD of at least three independent experiments [22].

### Liver function

Serum was isolated from whole blood by centrifuging (5000 rpm, 20 mins) at room temperature after coagulation. An automatic biochemical meter (CX7, Beckman Coulter, USA) was used to detect serum alanine aminotransferase (ALT) and aspartate transaminase (AST) levels.

### TUNEL staining

Briefly, the paraffin-embedded tissue sections were deparaffinized and dehydrated. A portion of 50 μl of TUNEL reaction mixture was added to tissue sections. Then, on each slide, 50 μl of conversion agent (POD) and 50–100 μl of substrate solution were added successively, and slides were incubated for 10 min at room temperature. Slides were then dehydrated, cleared and mounted for confocal microscopy observation. A positive signal was defined as the presence of distinct fluorescence staining within the nuclei 4′,6-diamidino-2-phenylindole (DAPI).

### Immunohistochemistry staining

All tissues were fixed in 4% paraformaldehyde for 36 h immediately after surgical resection and were embedded in paraffin. The tissue sections (5 μm) were heated, dewaxed and rehydrated by immersion in dimethylbenzene and a graded ethanol series. For immunohistochemistry, an antigen-retrieval technique consisting of heating in a citrate water bath was performed before dewaxing and rehydration. Endogenous peroxidase activity was blocked by incubation with 3% hydrogen peroxide, and nonspecific binding was blocked using 5% bovine serum albumin (BSA) at room temperature. Finally, specimens were incubated overnight with antibodies against PCNA (CST, Prod# 13110S, 1:200), and the sections were stained with diaminobezidin(DAB) the next day.

#### Ethynyl-2′-deoxyuridine cell proliferation assays

Cell proliferation was assessed by the incorporation of 5-ethynyl-2′-deoxyuridine (Cell-Light™ EdU In Vitro Imaging Kit, Prod# C10310, RiboBio Co) into DNA according to the manufacturer’s instructions. Briefly, hepatocytes were administered with PNS as described, and EdU was added to the culture at a concentration of 100 nmol/L. According to the standard formaldehyde fixation protocol, the cells were permeabilized, fixed and incubated with the reaction cocktail for 30 min. The images of stained cells were captured by fluorescence microscopy. Values represent the mean ± SD of at least three independent experiments.

### Western blotting

Total proteins were extracted from mouse liver and primary hepatocytes by RIPA Buffer (Abcam, Prod# ab156034) containing a protease inhibitor cocktail (Beyotime, Prod# P1005). A BCA Protein Assay Kit (CST, Prod#7780) was used to quantify the protein concentration in the supernatant, and lysates were then mixed and heated with Sample Loading Buffer (Beyotime, Prod# P0015A) at 100 °C for 10 min. Samples were separated by SDS-PAGE gels of different concentrations and were then transferred to a 0.2 μm PVDF membrane (ROCHE, Prod# 03010040001). The membranes were blocked with 5% nonfat milk (Bio-Rad, USA) for 60 min at room temperature and incubated overnight at 4 °C with primary antibodies against PI3K antibodies (cat. no. ab32089; 1:1000; Abcam Inc.); p-PI3K antibodies (cat. no. 4228; 1:1000; CST Inc.); AKT (cat. no. ab38449; 1:1000 dilution; Abcam Inc.); p-AKT (cat. no. ab38449; 1:1000; Abcam Inc.); mTOR (cat. no. ab2732; 1:1000; Abcam Inc.); mTOR (cat. no. ab109268; 1:1000; Abcam Inc.) GADPH (cat. no. ab8245; 1:1000 Abcam Inc.); Bad (cat. no. ab32445; 1:1000 Abcam Inc.); and p-Bad (cat. no. ab129192; 1:1000 Abcam Inc.), which was followed by incubation with horseradish peroxidase-conjugated secondary antibodies (ZSGB-BIO, Inc., China). Membranes were washed three times, incubated with HRP-conjugated secondary antibodies (ABclonal, Prod# AS003, AS014) at a dilution of 1:8000 for 60 min the next day and detected by a ChemiDoc™ system (Bio-Rad, USA) via Western-Bright ECL kit (Advansta, USA). β-actin was analyzed as a loading control.

### Liver/body weight ratio

The liver/body weight ratio, also known as the liver regeneration index, was calculated by the equation: A/B, in which A stands for liver weight after regeneration, while B stands for body weight.

### Annexin V-FITC/PI staining

We detected apoptosis in primary mouse hepatocytes by flow cytometry (FCM) using Annexin V-FITC/PI double staining. The cells were harvested and washed with PBS and incubated in binding buffer containing Annexin V-FITC and PI for 15 min at 37 °C in the dark. Cells were then analyzed using FCM (FACS CantoTM, BD, CA, USA). FlowJo software version 7.6.1 (Ashland, OR, USA) was used to count the number of apoptotic cells per sample.

### Statistical analysis

All values are expressed as mean ± SD. Data were analyzed using an unpaired two-tailed Student’s t-test or one-way analysis of variance with a post-hoc test. SPSS 22.0 software was applied in all statistical analyses. A *P* value less than 0.05 was required for results to be considered statistically significant.

## Results

### PNS promoted primary mouse hepatocyte proliferation in a dose-dependent manner and activated the PI3K/AKT/mTOR signaling pathway in primary mouse hepatocytes

**Fig. 1:**
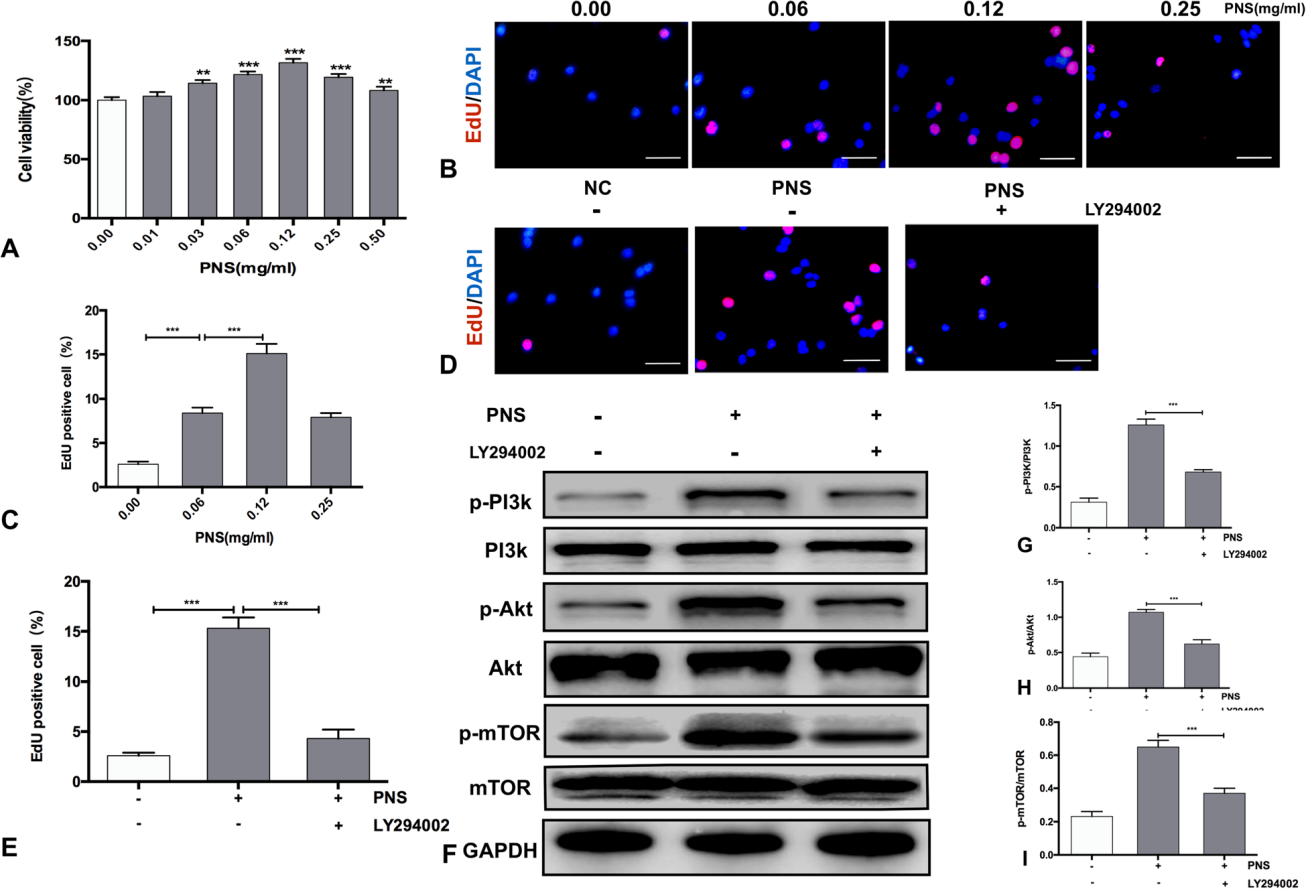
PNS promoted primary mouse hepatocyte proliferation via the PI3K/AKT/mTOR signaling pathway. The primary mouse hepatocytes were treated with a range of concentrations of PNS for 24 h. Cells were preincubated with or without 10 μM LY294002 for 1 h and were then treated with 0.12 mg/ml PNS for 24 h. (**a**) The cell viability was determined by CCK-8 Kits. (**b**) Primary mouse hepatocytes were pretreated with 0.00, 0.06, 0.12 and 0.25 mg/ml PNS for 24 h and EdU (pink) staining was measured (magnification: × 400, Scale bars represent 50 μm). (**c**) Quantification of EdU-positive cells. (**d**) Representative immunofluorescent images of primary mouse hepatocyte proliferation measured by EdU staining (magnification: × 400, Scale bars represent 50 μm). (**e**) Quantification of EdU-positive cells. (**f**) Total and phosphorylated protein levels of PI3K, AKT and mTOR in primary mouse hepatocytes. Values represent the mean ± SEM of 3 independent experiments. (**g**)(**h**)(**i**) Bar graph shows the quantification of total and phosphorylated protein levels of PI3K, AKT and mTOR. Values represent the mean ± SD of at least three independent experiments. **P < 0.01, *** *P* < 0.001

To test the effect of PNS on primary mouse hepatocytes, primary mouse hepatocytes were treated with PNS at concentrations ranging from 0.01 to 0.50 mg/ml for 24 h. PNS gradually increased primary mouse hepatocyte proliferation in a dose-dependent manner, peaking at 0.12 mg/ml. Then, the proliferative effect of PNS decreased with increasing PNS concentrations above 0.12 mg/ml (*P* < 0.05) (Fig. 1A). These results were similar to that of the EdU assay, in which the proliferative effect of PNS peaked at the 0.12 mg/ml treatment with 15.3% EdU-positive hepatocytes (Fig. 1B-C).

To examine whether PNS could activate AKT/mTOR in primary mouse hepatocytes, we added the PI3K/AKT inhibitor LY294002 to PNS-treated hepatocytes. The EdU staining showed that the proliferative effect of PNS was inhibited by LY294002 (*P* < 0.05) (Fig. 1D-E). The phosphorylated and total protein levels of PI3K, AKT, and mTOR were examined in hepatocytes treated with 0.12 mg/ml PNS with or without LY294002 by Western blotting. The results showed that PNS increased the phosphorylation of PI3K, AKT, and mTOR in primary mouse hepatocytes, and this effect was blocked by LY294002 (*P* < 0.05) (Fig. 1F-I).

### PNS promoted liver regeneration and activated the PI3K/AKT/mTOR signaling pathway in vivo

**Fig. 2:**
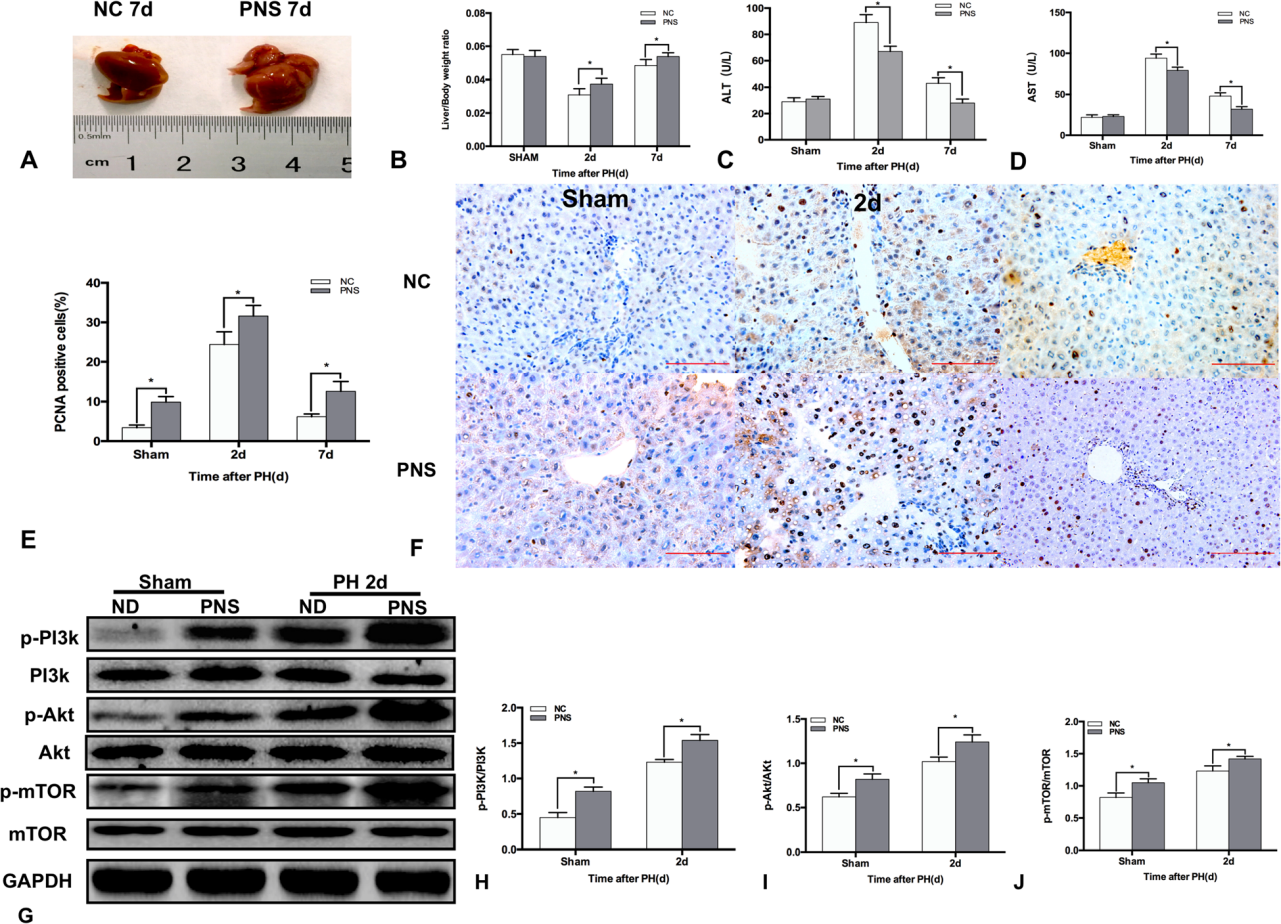
PNS activated the PI3K/AKT/mTOR signaling pathway during LR in vivo. C57BL/6 J mice were administered PNS 7 days before PH. (**a**) The regenerated liver in mice treated with or without PNS 7 days after PH. (**b**) Quantification of the relative ratio of liver weight/body weight in mice treated with or without PNS at 2 d and 7 d after PH. Serum concentrations of ALT (**c**) and AST (**d**) at 2 d and 7 d after PH. (**e**) Quantification of PCNA-positive cells in mice treated with or without PNS at 2 d and 7 d after PH. (**f**) Representative liver sections stained with PCNA in mice treated with or without PNS at 2 d and 7 d after PH (original magnification: × 200, scale bars represent 100 μm). (**g**) Total and phosphorylated protein levels of PI3k, AKT and mTOR in mice treated with or without PNS at 2 d after PH. (H)(I)(J) Bar graph shows the quantification of the total and phosphorylated protein levels of PI3k, AKT and mTOR. Values represent the mean ± SD of at least three independent experiments. * *P<*0.05

We determined whether PNS caused a proliferative effect in hepatocytes in vivo. The regenerated liver was larger 7 d after PH with PNS treatment (Fig. 2A). The liver/body weight ratios were higher at both 2 d and 7 d after PH with PNS treatment (*P* < 0.05) (Fig. 2B). The ALT and AST levels in serum are widely used biochemical markers for hepatic function. The serum ALT and AST levels were lower at both 2 d and 7 d after PH with PNS treatment (*P* < 0.05) (Fig. 2C-D). The PCNA staining showed that the proliferation of hepatocytes in vivo was higher in PNS group with or without PH (*P* < 0.05) (Fig. 2E-F). Western blotting showed that the phosphorylated protein levels of PI3K, AKT, and mTOR were higher in mice with PNS treatment than those of NC mice (*P* < 0.05) (Fig. 2G-J).

### PNS inhibited hepatocellular apoptosis during LR in mice through AKT/bad signaling

**Fig. 3:**
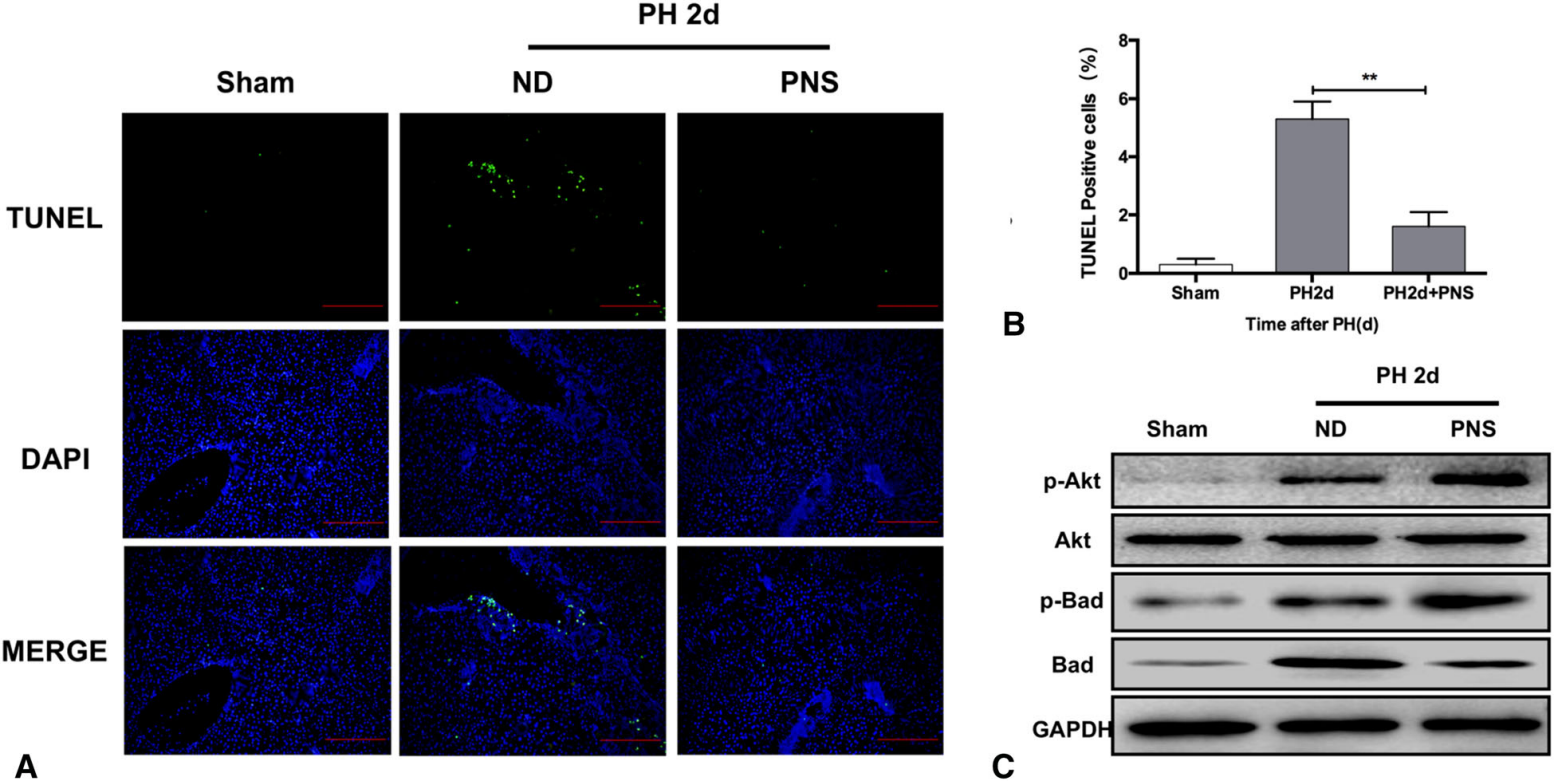
PNS inhibited cellular apoptosis during LR in vivo via the AKT/Bad pathway. (**a**) Representative liver sections stained by TUNEL in mice treated with or without PNS at 2 d after PH. (**b**) Quantification of TUNEL-positive cells in mice treated with or without PNS at 2 d after PH. (**c**) Total and phosphorylated protein levels of AKT and Bad in mice treated with or without PNS at 2 d after PH. Values represent the mean ± SD of at least three independent experiments; ** *P<*0.01

We determined whether PNS had an extra protective effect during LR. The TUNEL staining showed that cellular apoptosis was inhibited in mice with PNS treatment (*P* < 0.05) (Fig. 3A-B). The phosphorylated protein levels of AKT and Bad increased in PNS group (*P* < 0.05) (Fig. 3C).

### PNS alleviated hypoxia-induced hepatocellular apoptosis through AKT/bad signaling in vitro

**Fig. 4:**
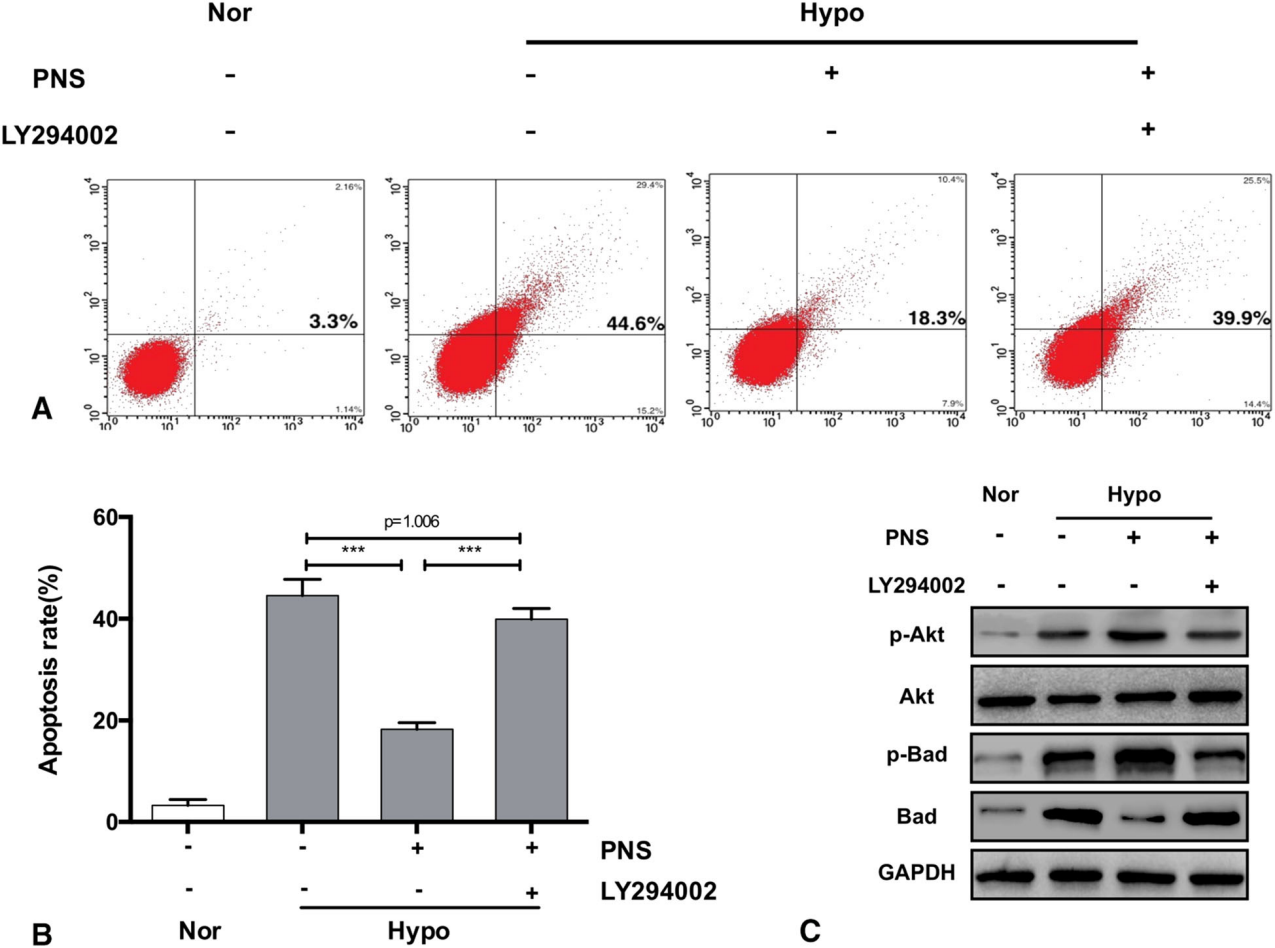
PNS inhibited hypoxia-induced cellular apoptosis via the AKT/Bad pathway in vitro. Primary mouse hepatocytes were preincubated with or without 10 μM LY294002 for 1 h and were then treated with 0.12 mg/ml PNS for 6 h before being subjected to hypoxia for 24 h. (**a**) The effect of a PI3K inhibitor on the protective activity of PNS against hypoxia-induced apoptosis in primary mouse hepatocytes was determined using Annexin V/PI staining by flow cytometry. (**b**) Quantification of (**a**). (**c**) Total and phosphorylated protein levels of AKT and Bad in primary mouse hepatocytes. Values represent the mean ± SD of at least three independent experiments; *** *P<*0.001

Annexin V/PI staining showed that the hypoxia-induced apoptosis rate decreased in primary hepatocytes treated with PNS comparing to that of hepatocytes without PNS treatment. However, LY294002 blocked the inhibitory effect of PNS on apoptosis induced by hypoxia in hepatocytes (Fig. 4A-C). These results indicated that the protective effect of PNS on hepatocytes involved the activation of PI3K/AKT/Bad pathways.

## Discussion

PNS is a species of the genus *Panax* and have extensive pharmacological activities in the treatment of various diseases, such as cardiovascular, nervous and anticancer, anti-atherosclerotic and other biological activities [23]. PNS are active components with different dammarane-type saponins [5]. *Notoginsenoside R1* is a unique component in PNS, which contributed to the amelioration of myocardial cells apoptosis and myocardial ischemia/reperfusion injury via inhibiting the activation of TAK1/NF-κB pathways [24]. Additionally, *Notoginsenoside R1* exerts protective effects through the enhancement of beta-amyloid protein degradation and maybe is a potential candidate for AD treatment [25]. For the effective treatment of cerebrovascular diseases and improves the cognitive and sensorimotor deficits, *Ginsenoside Rb1* as also a major active component in PNS has the function on the modulation of the AKT/mTOR signaling pathway and down-regulation of caspase-3 in rats subjected to phototrombic stroke [26]. It was indicated that the *ginsenoside Rh4* could trigger apoptosis by activating the ROS/JNK/p53 pathway in colorectal cancer cells [27]. In general, these components of PNS have various biological effects, such as antioxidant, anti-inflammatory, antiaging, neuroprotective, and anti-apoptosis and this prompted us to investigate the overall effects of PNS on LR and explore related molecular mechanisms. In the current study, we demonstrated that administration of PNS can promote LR and improve liver function after PH in mice through PI3K/AKT/mTOR pathway, thus such protective mechanism might mainly be induced by *Ginsenoside Rb1.* In our study, the results demonstrated that PNS at concentrations varying from 0.03 to 0.5 mg/ml promoted cell proliferation, which peaked at a concentration of 0.12 mg/ml in primary mouse hepatocytes; thus, we chose this concentration (0.12 mg/ml) for further study.

During LR, organisms developed complex protective mechanisms to survive in a challenging environment. Stress response proteins and pathways were involved in these mechanisms, including membrane transporters, protein chaperones, antioxidant enzymes, growth factors, and signaling pathways and transcriptional factors [28]. PI3K/AKT has long been considered a major pathway that promotes cell proliferation and prevents cellular apoptosis [29]. AKT phosphorylation initiates the expression of proteins involved in regulating proliferation and apoptosis in cells [30]. Cell apoptosis and survival, protein synthesis, and autophagy were regulated by phosphorylated mTOR, which was initiated by AKT [31]. In A KT knockout mice, LR was severely impaired and mortality increased due to the impairment of the proliferative capacity of hepatocytes [32, 33]. In our study, the results showed that PNS at a concentration of 0.12 mg/ml significantly increased the expression level of phosphorylated PI3K, AKT and mTOR. Administration of LY294002 blocked the proliferative effect of PNS and the PI3K/AKT/mTOR pathway, suggesting that PNS promoted cell proliferation in primary hepatocytes through the PI3K/AKT/mTOR pathway. In vivo, the results showed that PNS promoted LR. The regenerated remnant was larger than that of the NC group at 7 days after PH. The circulating levels of ALT and AST decreased with PNS treatment, indicating that PNS ameliorated liver injury induced by PH [34]. PCNA staining showed that PNS administration promoted cellular proliferation in regenerated liver. In our study, the phosphorylated levels of PI3K, AKT and mTOR increased at 2 days after PH, which is in accordance with a previous study [35], and the PI3K/AKT/mTOR pathway was elevated with administration of PNS.

TUNEL staining showed that apoptosis of hepatocytes increased at 2 days after PH, which is consistent with a previous study [36]. PNS significantly relieved cellular apoptosis after PH. Bad has been confirmed to be a downstream target of AKT in preventing cellular apoptosis [37]. Bad, which is a member of the Bcl-2 family, is able to combine with antiapoptotic Bcl-2 or Bcl-xL to form a complex that promotes apoptosis [38]. Previous studies have suggested that phosphorylation of Bad by AKT resulted in cell survival [39]. In our study, the results showed that p-Bad increased while Bad decreased after PNS administration. In vitro, we cultured primary hepatocytes in a hypoxic incubator to simulate the hypoxic state that hepatocytes undergo during LR. A previous study showed that AKT/Bad was activated during hypoxia [40]. Our results showed that PNS ameliorated the cellular apoptosis induced by hypoxia, and the AKT/Bad pathway was activated. However, these effects of PNS could be abolished by treatment with LY294002. In summary, the antiapoptotic effects of PNS during hypoxia were related to the activation of the AKT/Bad cell survival pathway.

## Conclusions

**Fig. 5:**
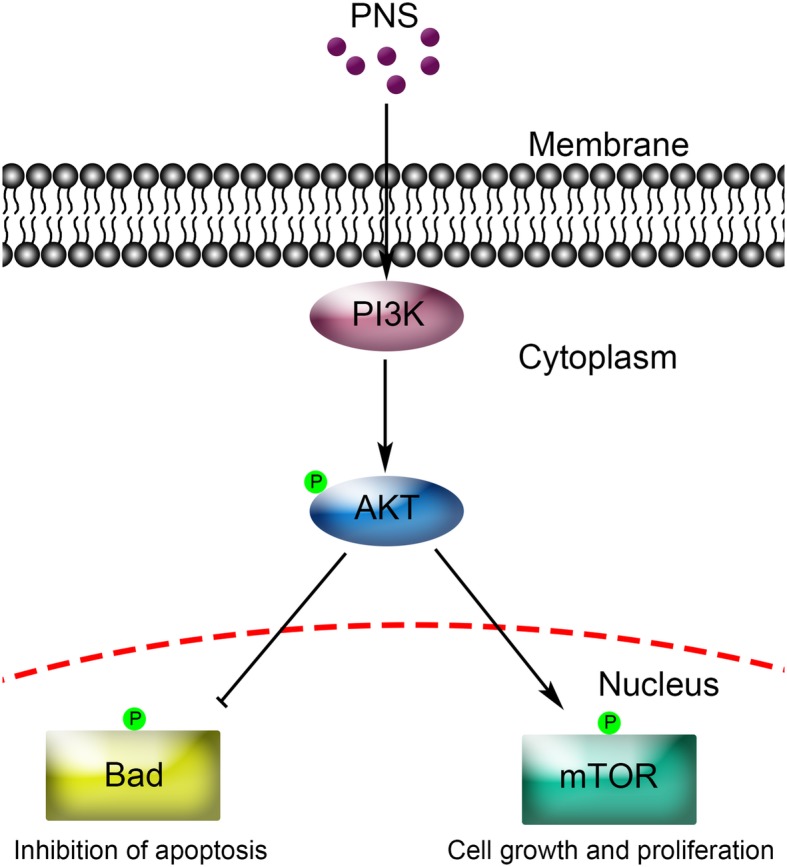
A schematic model of upregulated PI3K/AKT/ mTOR cell proliferation pathway and upregulated AKT/Bad cell survival pathway by PNS in primary mouse hepatocytes

The present study verified that PNS promoted hepatocyte proliferation during LR and protected hepatocytes against PH-induced apoptosis through the initiation of the PI3K/AKT/mTOR pathway and the PI3K/AKT/Bad pathway (as summarized in Fig. 5). The main findings of this study provided direct experimental evidence that PNS treatment could be a new approach to promote the regenerative capacity of the liver and accelerate the recovery of liver function in patients who have suffered from hepatic injuries caused by major hepatectomy.

## Data Availability

The datasets used and/or analysed during the current study are available from the corresponding author on reasonable request.

## Abbreviations

AKT: Protein kinase B
ALT: Anine aminotransferase
AST: Aspartate transaminase
BSA: Bovine serum albumin
DAB: diaminobezidin 3
DAPI: 4′,6-diamidino-2-phenylindole
EdU: 5-ethynyl-2′-deoxyuridine
HBSS: Hanks balanced salt solution
IVC: Inferior vena cava
LR: Liver regeneration
mTOR: Mammalian target of rapamycin
NC: Normal control
PCNA: Proliferating cell nuclear antigen
PH: Partial hepatectomy
PI3K: Phosphatidylinositol-3-kinase
PNS: *Panax notoginseng* saponins
SPF: Specific pathogen free
